# Survey on Specialty Preference and Work-Life Balance among Residents of Japanese Red Cross Hospitals

**DOI:** 10.31662/jmaj.2019-0013

**Published:** 2020-04-07

**Authors:** Anna Nakayasu, Michiko Kido, Keiichi Katoh, Yukio Homma

**Affiliations:** 1 Department of Obstetrics and Gynecology, Japanese Red Cross Medical Center, Tokyo, Japan; 2 Department of Anesthesiology, Japanese Red Cross Medical Center, Tokyo, Japan; 3 President, Japanese Red Cross Medical Center, Tokyo, Japan

**Keywords:** Work-life balance, specialty selection, lifestyle, medical education, career, maldistribution of physicians, Japanese health care

## Abstract

**Introduction:**

The paucity and maldistribution of physicians among various specialties are key issues facing the Japanese health care system. Studies have shown that young physicians place more emphasis on work-life balance while selecting their specialty and that they prefer controllable lifestyle (CL) specialties over noncontrollable lifestyle (NCL) specialties. As this may be a cause of maldistribution, we investigated the relationship between views on work-life balance and specialty selection among young physicians in Japan.

**Methods:**

An online questionnaire was sent to 1451 residents (postgraduate years 1-5) at 60 Japanese Red Cross hospitals across Japan.

**Results:**

In all, 226 physicians responded (response rate: 15%), with 21% in CL and 74% in NCL specialties. When compared with NCL specialties, CL specialties had less overtime (43% vs. 16%, *p* = 0.001), considered life to be more important than work (26% vs. 15%, *p* = 0.018), and were more likely to give precedence to work-life balance over medical interest while choosing their specialty (49% vs. 30%, *p* < 0.001). Furthermore, physicians were more likely to change their choice of specialty, contrary to their professional interest, because of social reasons (49% vs. 26%, *p* = 0.007).

**Conclusions:**

Our study suggests that young physicians in CL specialties have better working hours and place more emphasis on work-life balance while choosing their specialty compared with those in NCL specialties. The increase in the number of physicians in CL specialties is likely attributable to the growing preference for an optimal work-life balance among young physicians; this seems to have increased the maldistribution of physicians among various specialties. Institutional mechanisms to support the lifestyle of physicians (especially in NCL specialties) are required to provide a balanced medical service in Japan.

## Introduction

Progressive population aging in Japan has increased the demand for health care services, which calls for an increase in medical personnel. However, the paucity of physicians has been an issue in Japan for the past few decades. The number of physicians per 1000 people in Japan is one of the lowest among the OECD (Organisation for Economic Co-operation and Development) countries ^(1)^. Moreover, the maldistribution of physicians among various specialties is a key challenge that may further aggravate the shortage of physicians. Understanding the preferences of young physicians is key to future planning for a more balanced medical system.

Previous studies have indicated that young physicians tend to opt for specialties that allow an optimal work-life balance ^(2), (3)^. In a study, about 30% of graduate medical trainees claimed that their career plans were influenced by life events ^(4)^. In pioneering work published in 1990, Schwartz et al. categorized medical specialties into “controllable lifestyle (CL)” specialties (such as anesthesiology, dermatology, emergency medicine, neurology, ophthalmology, otolaryngology, pathology, psychiatry, and radiology) and “noncontrollable lifestyle (NCL)” specialties (such as internal medicine, family practice, pediatrics, obstetrics/gynecology, and surgery) ^(5)^. Another study by Schwartz et al. documented an increase in the number of medical students opting for CL specialties and a decrease in the number of those opting for NCL specialties ^(6)^. A study between 1996 and 2003 showed that the proportion of medical students who opted for CL specialties increased from 18% to 36% among women and from 28% to 45% among men, which suggests the absence of any gender-specific trend in the selection ^(7)^. In another study conducted in 2013, the so-called R.O.A.D. (radiology, ophthalmology, anesthesia, and dermatology) specialties, which are reportedly associated with the best lifestyle, ranked as the top specialties preferred by medical students ^(8)^. These preferences seem natural, considering evidence of the close relationship between work-life balance and the incidence of burnout or even suicide among physicians ^(9)^.

It is expected that a similar trend should be seen in Japan, although no study to date has investigated the relationship between choice of specialty and views pertaining to work-life balance. In this study, we sought to understand the relationship between the views of young Japanese physicians about work-life balance, specialty selection, and career building by novel data on the following: working hours of various specialties, trend in specialties chosen, and young physicians’ values about lifestyle. Our findings may help inform interventions to resolve the maldistribution of Japanese physicians among various specialties.

## Materials and Methods

### Participants

We conducted a cross-sectional survey of resident physicians working at the Japanese Red Cross network hospitals. The network is composed of 92 hospitals at the core of local health care services. Of these facilities, 60 have residency programs. In the Japanese postgraduate education system, postgraduate year (PGY) 1-2 physicians (junior residents) rotate through various specialties on a monthly basis, and PGY 3-5 physicians (senior residents) choose and train in a specific specialty. A questionnaire was sent to 1451 young physicians of PGY 1-5 (967 junior residents and 484 senior residents) working at the 60 hospitals.

### Ethics approval and consent to participate

The study was approved by the clinical research ethics committee of the Japanese Red Cross Medical Center on January 17, 2018, Reference No. 852. The respondents gave their consent to participate on sending the answers to the questionnaire, under the condition that data were shown in a form where individuals could not be specified.

### Questionnaire

The questionnaire (Appendix 1) along with an invitation letter was directly sent to the residents, who responded to the questionnaire via a Google form link. The questionnaire included (1) basic information such as PGY, hometown, location of university of graduation, and location of workplace; (2) preference and choice of specialties; (3) opinions on shortage of physicians in rural Japan; and (4) opinions about work-life balance and current working conditions.

### Data analysis

Categorization of the specialties is based on the Japanese Medical Specialty Board ^(10)^, an NPO that standardizes board specializations across the nation. The specialties were divided into three categories as proposed by Schwartz et al.: CL specialties; NCL specialties; and others ^(5), (6)^. Urology, neurosurgery, orthopedics, and plastic surgery were categorized as surgery. Between-group differences were assessed using the Fisher test; *p* values less than 0.05 were considered to be statistically significant. All statistical analyses were performed with EZR (Saitama Medical Center, Jichi Medical University, Saitama, Japan), which is a graphical user interface for R (The R Foundation for Statistical Computing, Vienna, Austria) ^(11)^.

## Results

### Study group

Only 226 out of 1451 residents (15%) responded to the survey. The distribution of respondents was 83 (40%) PGY 1, 53 (25%) PGY 2, 25 (12%) PGY 3, 25 (12%) PGY 4, and 23 (11%) PGY 5. The preference for specialties was answered by 221 respondents, as shown in Table 1. NCL specialties were chosen by 74% of the respondents, whereas 21% opted for CL specialties. As a reference, the proportion of specialties chosen by PGY 3 physicians in the Japan Medical Specialty Board matching program is given in the right column ^(10)^. The distribution of specialties in our sample is similar to that in the Board matching program.

**Table 1.** Specialty Preference.

		Study group		Reference	
		No.	%	No.	%
NCL	Internal medicine	79	(36)	2527	(32)
	General surgery	23	(10)	767	(10)
	Pediatrics	25	(11)	526	(7)
	Ob/gyn	19	(9)	410	(5)
	Urology	3	(1)	247	(3)
	Neurosurgery	3	(1)	214	(3)
	Orthopedics	9	(4)	516	(7)
	Plastic surgery	3	(1)	153	(2)
CL	Otolaryngology	5	(2)	238	(3)
	Radiology	8	(4)	244	(3)
	Dermatology	4	(2)	254	(3)
	Psychiatry	4	(2)	392	(5)
	Emergency medicine	7	(3)	234	(3)
	Anesthesiology	15	(7)	457	(6)
	Ophthalmology	2	(1)	288	(4)
	Pathology	2	(1)	101	(1)
Others	Laboratory medicine	0	(0)	4	(0)
	Rehabilitation	0	(0)	66	(1)
	Family medicine	2	(1)	153	(2)
	Undecided	8	(4)	0	(0)
	TOTAL	221		7791	
					
	NCL total	164	(74)	5360	(69)
	CL total	47	(21)	2208	(28)

NCL: noncontrollable lifestyle; CL: controllable lifestyle; Ob/gyn: Obstetrics/gynecology.

### Working hours depending on specialty

The majority of residents (58%) worked for 4-5 night shifts per month, regardless of the chosen specialty (Table 2). The overall percentage of those who worked for more than 6 night shifts per month was 10%. The number of night shifts was not significantly different between the NCL and CL specialties (*p* = 0.38), although the frequency of >6 night shifts per month in NCL specialties was higher than that in CL specialties.

**Table 2.** Working Hours Depending on Specialty.

		Number of night shifts per month						Hours of overtime per month					
		1-3/month		4-5/month		More than 6/month		Less than 45 h^*^		45-99 h		More than 100 h^**^	
		No.	%	No.	%	No.	%	No.	%	No.	%	No.	%
NCL	Internal medicine	36	(46)	36	(46)	6	(8)	13	(17)	42	(55)	22	(29)
	General surgery	5	(22)	17	(74)	1	(4)	4	(17)	8	(35)	11	(48)
	Pediatrics	4	(16)	16	(64)	5	(20)	4	(16)	16	(64)	5	(20)
	Ob/gyn	3	(16)	12	(63)	4	(21)	2	(11)	15	(79)	2	(11)
	Urology	1	(33)	1	(33)	1	(33)	0	(0)	1	(33)	2	(67)
	Neurosurgery	1	(33)	2	(67)	0	(0)	0	(0)	2	(67)	1	(33)
	Orthopedics	2	(22)	7	(78)	0	(0)	2	(22)	7	(78)	0	(0)
	Plastic surgery	0	(0)	3	(100)	0	(0)	1	(50)	1	(50)	0	(0)
CL	Otolaryngology	2	(40)	3	(60)	0	(0)	3	(60)	1	(20)	1	(20)
	Radiology	5	(63)	3	(38)	0	(0)	4	(50)	4	(50)	0	(0)
	Dermatology	2	(50)	2	(50)	0	(0)	2	(50)	1	(25)	1	(25)
	Psychiatry	2	(67)	1	(33)	0	(0)	1	(25)	3	(75)	0	(0)
	Emergency medicine	0	(0)	6	(86)	1	(14)	1	(14)	4	(57)	2	(29)
	Anesthesiology	6	(40)	9	(60)	0	(0)	7	(47)	6	(40)	2	(13)
	Ophthalmology	0	(0)	1	(50)	1	(50)	1	(50)	0	(0)	1	(50)
	Pathology	1	(50)	1	(50)	0	(0)	1	(50)	1	(50)	0	(0)
Others	Laboratory medicine	0	(0)	0	(0)	0	(0)	0	(0)	0	(0)	0	(0)
	Rehabilitation	0	(0)	0	(0)	0	(0)	0	(0)	0	(0)	0	(0)
	Family medicine	1	(50)	0	(0)	1	(50)	0	(0)	2	(100)	0	(0)
	Undecided	1	(13)	6	(75)	1	(13)	0	(0)	5	(63)	3	(38)
	TOTAL	72	(33)	126	(58)	21	(10)	46	(21)	119	(55)	53	(24)

	NCL total	52	(32)	94	(58)	17	(10)	26	(16)	92	(57)	43	(27)
	CL total	18	(39)	26	(57)	2	(4)	20	(43)	20	(43)	7	(15)
		*p* = 0.38						*p* = 0.001					

*45 h: upper limit of overtime hours per month under the Labor Standards Act of Japan. **100 h: upper limit of overtime hours per month in the event that the employer has entered into a written agreement.

The most commonly reported duration of overtime (55%) was 45-99 h per month (Table 2). NCL specialties were more likely to be associated with >100 h of overtime per month as compared with CL specialties (27% vs. 15%, respectively). However, CL specialties were more likely to involve <45 h of overtime per month as compared with NCL specialties (43% vs. 16%, respectively). CL specialties were associated with a significantly lower duration of overtime as compared with NCL specialties (*p* = 0.001).

### Views on work-life balance

The questionnaire contained two items pertaining to views on work-life balance (Table 3). The relative importance of work versus life was situational for most respondents (70%), followed by life (18%) and work (12%). Work and life were viewed as even (both 15%) by respondents in NCL specialties, whereas life was significantly more important (26% vs. 2%) for respondents in CL specialties. This suggests that those in CL specialties gave precedence to life over work significantly as compared with those in NCL specialties (*p* = 0.018).

**Table 3.** Opinions on Work-Life Balance.

		Emphasis on work or life						Emphasis on medical interest or social reasons in specialty selection					
		Depends		Life		Work		Medical		Social		Neither	
		No.	%	No.	%	No.	%	No.	%	No.	%	No.	%
NCL	Internal medicine	45	(58)	17	(22)	16	(21)	40	(51)	24	(31)	14	(18)
	General surgery	20	(87)	1	(4)	2	(9)	10	(43)	4	(17)	9	(39)
	Pediatrics	20	(83)	3	(13)	1	(4)	10	(40)	7	(28)	8	(32)
	Ob/gyn	13	(68)	2	(11)	4	(21)	12	(63)	5	(26)	2	(11)
	Urology	3	(100)	0	(0)	0	(0)	0	(0)	2	(67)	1	(33)
	Neurosurgery	2	(67)	0	(0)	1	(33)	3	(100)	0	(0)	0	(0)
	Orthopedics	7	(78)	1	(11)	1	(11)	2	(22)	6	(67)	1	(11)
	Plastic surgery	2	(67)	1	(33)	0	(0)	1	(33)	1	(33)	1	(33)
CL	Otolaryngology	4	(80)	1	(20)	0	(0)	2	(40)	2	(40)	1	(20)
	Radiology	7	(88)	1	(13)	0	(0)	1	(13)	5	(63)	2	(25)
	Dermatology	3	(75)	1	(25)	0	(0)	1	(25)	1	(25)	2	(50)
	Psychiatry	3	(75)	1	(25)	0	(0)	1	(33)	1	(33)	2	(67)
	Emergency medicine	5	(71)	1	(14)	1	(14)	1	(14)	2	(29)	4	(57)
	Anesthesiology	9	(60)	6	(40)	0	(0)	2	(13)	9	(60)	4	(27)
	Ophthalmology	1	(50)	1	(50)	0	(0)	0	(0)	2	(100)	0	(0)
	Pathology	2	(100)	0	(0)	0	(0)	0	(0)	1	(50)	1	(50)
Others	Laboratory medicine	0	(0)	0	(0)	0	(0)	0	(0)	0	(0)	0	(0)
	Rehabilitation	0	(0)	0	(0)	0	(0)	0	(0)	0	(0)	0	(0)
	Family medicine	2	(100)	0	(0)	0	(0)	2	(100)	0	(0)	0	(0)
	Undecided	5	(63)	2	(25)	1	(13)	4	(50)	4	(50)	0	(0)
TOTAL		153	(70)	39	(18)	27	(12)	92	(42)	76	(35)	52	(24)

	NCL total	112	(69)	25	(15)	25	(15)	78	(48)	49	(30)	36	(22)
	CL total	34	(72)	12	(26)	1	(2)	8	(17)	23	(49)	16	(34)
		*p* = 0.018						*p* < 0.001					

Factors that influenced the choice of specialty were debatable between medical interest (42%) and social reasons (35%). In addition, residents in CL specialties tended to place greater emphasis on social factors (49%) as compared with those in NCL specialties (30%). Those in CL specialties tended to place more emphasis on social reasons and less on medical interest while opting for a specialty; the between-group difference in this respect was statistically significant (*p* < 0.001).

We asked the respondents whether they changed their choice of specialty, contrary to their professional interest, because of social reasons (Table 4). Overall, 30% answered in the affirmative. On subgroup analysis, respondents in CL specialties were significantly more likely to answer yes compared with those in NCL specialties (49% vs. 26%, *p* = 0.007).

**Table 4.** Experience in Changing Specialty Selection Due to Social Reasons despite Having Medical Interest.

	No		Yes		Neither	
	No.	%	No.	%	No.	%
NCL total	93	(57)	42	(26)	28	(17)
CL total	16	(34)	23	(49)	8	(17)
	*p* = 0.007					

## Discussion

Previous studies have shown an increasing trend in the number of young physicians who opt for the so-called CL specialties ^(2), (3), (6), (8)^. In this nationwide survey of young physicians, the results show a clear distinction between specialties with respect to the number of working hours and the emphasis of respondents on work or life. The observed trend is in line with past studies that categorized medical specialties into CL or NCL. It appears that young physicians find an adequate balance between their medical interest and work-life balance while exercising their choice of specialty. This means that the number of young physicians who aspire to pursue a certain specialty is limited by the extent to which that specialty allows control over working conditions and lifestyle.

With respect to working conditions, our data show that CL specialties are more likely to involve less hours of overtime, and support the fact that CL specialties allow more controllable, flexible hours that physicians can use for themselves. In a study by Tait et al., the percentage of those satisfied with their work-life balance in NCL specialties was less than that in CL specialties ^(9)^. Moreover, in a study conducted among surgical residents, the number of hours worked per week was a significant predictor of burnout, low career satisfaction, and poorer quality of life ^(12)^.

In terms of views pertaining to work-life balance, those in CL specialties considered life to be more important than work and gave precedence to social reasons over medical interest while opting for a specialty. They were also more likely to change their choice of specialty, contrary to their professional interest, because of social reasons. From these results, we can assume that some young physicians in CL specialties may have aspired to opt for an NCL specialty but changed to a CL specialty because of lifestyle-related reasons. This is consistent with another study in which work-life balance was the major reason for physicians who initially considered a specialty but eventually rejected it ^(13)^.

If those in CL specialties tend to emphasize life and sacrifice medical interest to meet their social needs, and if more young physicians give precedence to life over work, the number of physicians in CL specialties is expected to increase, whereas the number in NCL specialties is expected to decrease.

Data from the Ministry of Health, Labour and Welfare show an increasing trend in the percentage of physicians in anesthesiology, radiology, psychiatry, and dermatology ^(3), (14)^. These specialties fall under the category of CL specialties, and according to our data, this trend may be attributable to the preference of physicians for an optimal work-life balance. The so-called NCL specialties (such as internal medicine, general surgery, pediatrics, obstetrics/gynecology, and neurosurgery), however, struggle to attract young physicians. Because these specialties involve longer working hours, physicians in these specialties are more likely to view work as more important than life. Here, we can see that CL specialties are more popular among the younger generation of physicians than NCL specialties. The importance of work-life balance has increased the preference for CL specialties. These results suggest that young physicians’ views regarding work-life balance may be related to the maldistribution of physicians among various specialties. A study of first-year physicians in Hungary also pointed out that the shortage of physicians in certain specialties may be improved through interventions related to income and working conditions ^(15)^.

To resolve this situation, efforts should be made to improve the work-life balance in all specialties, especially in the NCL specialties. This is also an ethical issue, considering that there may be too much workload on physicians in certain specialties. Countermeasures such as sharing tasks with other medical professionals, restriction of working hours, and a change in cultures that allow long hours are necessary to improve the lifestyle of physicians, especially in NCL specialties.

A major limitation of this study is the small sample size and the low response rate. The response rate was particularly low in specialties that typically have a fewer number of physicians. This may be because even though we were able to distribute the questionnaire to each hospital, we were unable to access the physicians and ensure that they answer the questionnaire, and the outcome was based solely on their good intentions. With respect to working conditions, those in PGY 1-2 are junior residents that rotate through various specialties; this implies that their working hours are not necessarily representative of the actual working conditions in that specialty. Next, the categorization into CL and NCL specialties is rather ambiguous. Although a number of studies have used this categorization ^(2), (5), (6), (15)^, it may represent an oversimplification. Not all physicians have similar working conditions even within the same specialty. Finally, we did not disaggregate our analysis by gender, which may also shape opinions about work-life balance. Although some studies have claimed a similar trend in both male and female physicians ^(7)^, others claim that female doctors place greater emphasis on work-life balance ^(16), (17)^.

### Conclusions

This study suggests that lifestyle tends to influence the choice of young physicians for a particular specialty. Those who view lifestyle as important are likely to select CL specialties. With an increase in young physicians who place greater emphasis on lifestyle, this trend may be one reason for the maldistribution of physicians among various specialties, and a warning that working styles must be improved in all specialties. The institutionalization of systems to support the lifestyle of physicians, especially in NCL specialties, is key to providing a balanced medical service while maintaining the freedom of occupational choice in Japan.

## Article Information

### Conflicts of Interest

None

### Acknowledgement

We thank all the hospitals of the Japanese Red Cross Society and the residents that participated in the survey. Special thanks to Dr. Yudai Izumo and Dr. Nobuyasu Awano of the Department of Pulmonary Medicine at the Japanese Red Cross Medical Center for their support in statistical analysis.

### Author Contributions

AN analyzed the survey results and was the main writer of the manuscript. MK, KK, and YH were major contributors in editing the manuscript. All authors contributed to the study conceptualization and design and have read and approved the final manuscript.

### Approval by Institutional Review Board (IRB)

The study was approved by the clinical research ethics committee of the Japanese Red Cross Medical Center on January 17, 2018, Reference No. 852. The respondents gave their consent to participate on sending the answers to the questionnaire, under the condition that data were shown in a form where individuals could not be specified.

## Supplement

APPENDIX1.
