# Supplementary material for: Survey on Specialty Preference and Work-Life Balance among Residents of Japanese Red Cross Hospitals
Source: JMA J. 2020 Apr 7;3(2):118–24. doi: 10.31662/jmaj.2019-0013 (PMC7590388; doi:10.31662/jmaj.2019-0013)
Supplement: Supplementary file 1 — APPENDIX1. [file 2433-3298-3-2-0118-s001.pdf]

APPENDIX1.

The questionnaire

The actual questionnaire was created in Japanese using Google form, and answers were collected online. The content of the questionnaire is as written in the methods section. Excerpt of questions regarding data included in the analysis are shown below translated into English.

1 What year of your graduate medical education training are you in?

(A) PGY 1

(B) PGY 2

(C) PGY 3

(D) PGY 4

(E) PGY 5

2 Which specialty are you in, or considering in going into?

(A) Internal Medicine

(B) General Surgery

(C) Pediatrics

(D) Ob/Gyn

(E) Urology

(F) Neurosurgery

(G) Orthopedics

(H) Plastic Surgery

(I) Otolaryngology

(J) Radiology

(K) Dermatology

(L) Psychiatry

(M) Emergency Medicine

(N) Anesthesiology

(O) Ophthalmology

(P) Pathology

(Q) Laboratory Medicine

(R) Rehabilitation

(S) Family Medicine

(T) Undecided

3 How many hours of overtime do you have per month?

(A) Less than 45 hours

(B) 45-79 hours

(C) 80-99 hours

(D) 100-149 hours

(E) 159-199 hours

(F) More than 200 hours

4 How many night shifts do you have per month?

(A) 1-3/month

(B) 4-5/month

(C) More than 6/month

5 (Question to physicians who changed their choice in specialty) What is the main reason you changed your specialty?

(A) Lack in medical interest

(B) Unsatisfied with residency program

(C) Advice from elder physicians

(D) Unsatisfied with salary

(E) Unsatisfied with Work-life balance

6 Which is more important, work or life?

(A) Work

(B) Depends on situation

(C) Life

7 Which is more important to you in choosing your specialty, social reasons such as residency programs and work-life balance, or medical interest?

(A) Social reasons

(B) Medical interest

(C) Neither

8 Have you changed your choice in specialty due to work-life balance although you had medical interest?

(A) Yes

(B) No

(C) Neither
